# Increasing Genome Sampling and Improving SNP Genotyping for Genotyping-by-Sequencing with New Combinations of Restriction Enzymes

**DOI:** 10.1534/g3.115.025775

**Published:** 2016-01-27

**Authors:** Yong-Bi Fu, Gregory W. Peterson, Yibo Dong

**Affiliations:** Plant Gene Resources of Canada, Saskatoon Research and Development Centre, Agriculture and Agri-Food Canada, Saskatoon, Saskatchewan S7N 0X2, Canada

**Keywords:** genotyping-by-sequencing, restriction enzyme combination, genome coverage, SNP genotyping, *in silico* analysis

## Abstract

Genotyping-by-sequencing (GBS) has emerged as a useful genomic approach for exploring genome-wide genetic variation. However, GBS commonly samples a genome unevenly and can generate a substantial amount of missing data. These technical features would limit the power of various GBS-based genetic and genomic analyses. Here we present software called IgCoverage for *in silico* evaluation of genomic coverage through GBS with an individual or pair of restriction enzymes on one sequenced genome, and report a new set of 21 restriction enzyme combinations that can be applied to enhance GBS applications. These enzyme combinations were developed through an application of IgCoverage on 22 plant, animal, and fungus species with sequenced genomes, and some of them were empirically evaluated with different runs of Illumina MiSeq sequencing in 12 plant species. The *in silico* analysis of 22 organisms revealed up to eight times more genome coverage for the new combinations consisted of pairing four- or five-cutter restriction enzymes than the commonly used enzyme combination *Pst*I + *Msp*I. The empirical evaluation of the new enzyme combination (*Hin*fI + *Hpy*CH4IV) in 12 plant species showed 1.7–6 times more genome coverage than *Pst*I + *Msp*I, and 2.3 times more genome coverage in dicots than monocots. Also, the SNP genotyping in 12 *Arabidopsis* and 12 rice plants revealed that *Hin*fI + *Hpy*CH4IV generated 7 and 1.3 times more SNPs (with 0–16.7% missing observations) than *Pst*I + *Msp*I, respectively. These findings demonstrate that these novel enzyme combinations can be utilized to increase genome sampling and improve SNP genotyping in various GBS applications.

Genotyping-by-sequencing (GBS) has emerged as a useful genomic approach for exploring genetic variation and performing association mapping on a genome-wide scale (Huang *et al.* 2009; Elshire *et al.* 2011; Fu and Peterson 2011; Poland and Rife 2012), thanks to the advances in next-generation sequencing technologies (Metzker 2010). The GBS approach is a combined one-step process of SNP marker discovery and genotyping through genome reduction with restriction enzymes (RE) (Altshuler *et al.* 2000; van Orsouw *et al.* 2007) and SNP calls with or without a sequenced genome (Elshire *et al.* 2011; Fu and Peterson 2011). This approach has displayed a major advantage of being rapid, high throughput, and cost-effective for genome-wide analysis of genetic variation and association mapping (Davey *et al.* 2011; Poland and Rife 2012; Fu *et al.* 2014). However, GBS usually samples a genome unevenly (Beissinger *et al.* 2013; Schilling *et al.* 2014) and can generate SNP data with a large proportion of missing observations across assayed samples (Marchini and Howie 2010; Rutkoski *et al.* 2013; Fu 2014). These technical features would limit the power of various GBS-based genetic and genomic analyses (Pool *et al.* 2010; Nielsen *et al.* 2011; Crawford and Lazzaro 2012).

Efforts have been made to recover information from GBS SNP data with missing observations. Several imputation methods based on row averages, row medians, and data correlation (Little and Rubin 1987; Horton and Kleinman 2007; Carpenter and Kenward 2013) have been applied to infer missing genotypes for a genetic analysis (Troyanskaya *et al.* 2001; Iwata and Jannink 2010; Weigel *et al.* 2010). Specific efforts to impute unordered SNP genotype data have also been made using regression-based methods such as Random Forest (Breiman 2001; Stekhoven and Bühlmann 2011) and principal component analysis-based tools (Stacklies *et al.* 2007) with the hope of improving genomic selection (Poland *et al.* 2012b; Rutkoski *et al.* 2013). These efforts help to regain some missing information, particularly from those datasets with fewer than 30% observations missing, but the information gain is less ideal (Fu 2014; Huang *et al.* 2014) as some GBS applications could generate up to 90% missing data in SNP genotyping (Elshire *et al.* 2011; Fu and Peterson 2011).

Research into efficient genome sampling by various REs for genome reduction has helped to develop different GBS protocols (Elshire *et al.* 2011; Fu and Peterson 2011; Peterson *et al.* 2012; Poland *et al.* 2012a; Sonah *et al.* 2013; Peterson *et al.* 2014). Specifically, Elshire *et al.* (2011) presented a GBS protocol using a methylation-sensitive RE, *Ape*KI, followed by the release of the two-enzyme (*Pst*I + *Msp*I) protocol by Poland *et al.* (2012a) and the double digest RADseq protocol by Peterson *et al.* (2012). The two-enzyme protocol has good technical merit and has gained popularity in application for generating high-density genetic maps (Poland *et al.* 2012a), but alternative protocols also exist (Andrews *et al.* 2014; Puritz *et al.* 2014). Overall, these efforts have enhanced the genome sampling of the GBS approach, particularly with the double-digest protocols (Peterson *et al.* 2012; Poland *et al.* 2012a). However, these GBS protocols may not have adequate genome sampling for high-density mapping, nor sufficiently reduce missing data (Beissinger *et al.* 2013; Schilling *et al.* 2014; Sims *et al.* 2014). Further efforts have been made to improve the GBS efficiency in genome sampling (De Donato *et al.* 2013; Heffelfinger *et al.* 2014; Schilling *et al.* 2014; Peterson *et al.* 2014), particularly with the use of more effective enzyme combinations (Hamblin and Rabbi 2014).

We conducted a search for better RE combinations for GBS applications with the goal of increasing genome sampling and improving SNP genotyping. Our search focused on the development of software, IgCoverage, for *in silico* evaluation of genomic coverage through GBS with an individual or a pair of REs on one sequenced genome, and had a larger scope with 70 RE combinations and 22 model organisms than the previous efforts (Poland *et al.* 2012a; Peterson *et al.* 2012). The specific objective of this search was to explore a new set of RE combinations for improving GBS applications through *in silico* analyses of 22 organisms and empirical evaluations in some plant species. It was our hope that this exploration would generate a list of candidate RE combinations for broader GBS application in different species, and provide some useful tools for further searches for specific RE combinations for GBS applications in a species of particular interest.

## Materials and Methods

**Figure 1:**
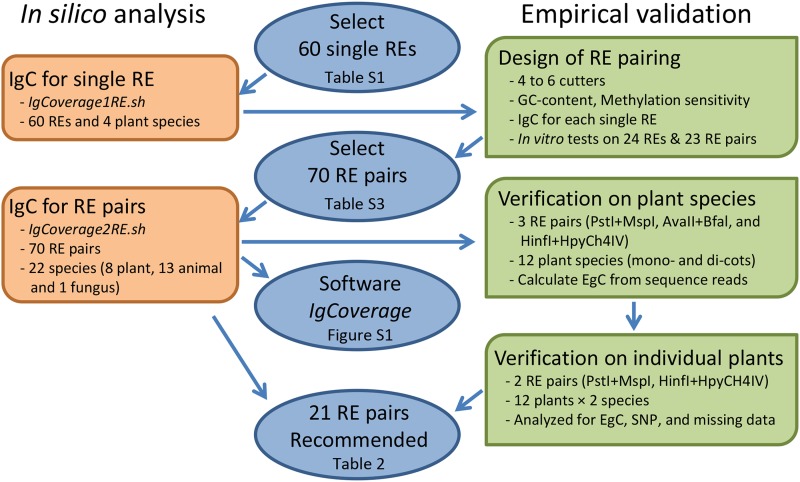
Procedures used to explore new REs for GBS application. A flow chart for exploring restriction enzyme (RE) pairs for a genome-by-sequencing (GBS) application to increase genome coverage of a species through *in silico* analysis and empirical validation. The genome coverage is measured by the proportion of the genome covered by a selected set of DNA fragments digested with a RE or RE pair. IgC and EgC are the genome coverages of a species estimated from *in silico* analysis and empirical validation, respectively. Two shell scripts (IgCoverage1RE.sh and IgCoverage2RE.sh) are part of software IgCoverage developed for this study.

Our search for new RE combinations with increased genome sampling for a GBS application was done through comparisons of genome coverages of a species obtained by the new RE combinations and the commonly used RE pair *Pst*I + *Msp*I, either from an *in silico* analysis or empirical validation. In this study, we defined the genome coverage as the proportional genome covered by DNA fragments digested by a RE or RE pair. To avoid confusion, we named IgC and EgC as the genome coverages of a species estimated from the *in silico* analysis and empirical validation, respectively. Specifically, the search had five major interactive components through *in silico* analysis and empirical evaluation (Figure 1). They are (C1) *in silico* analysis of IgC for single REs, (C2) designing RE pairs, (C3) *in silico* analysis of IgC for RE pairs, (C4) empirical verification of IgC for some RE pairs on plant species, and (C5) further empirical verification for some RE pairs on individual plants. The details of each major step are described below.

### C1: in silico analysis of IgC for individual REs

The *in silico* search for informative REs started with the selection of 60 REs (Supporting Information, Table S1) by considering the enzyme usage, recognition site and length, methylation sensitivity, active temperature, and unit cost. The majority of the selected enzymes were four- to six-cutters (or site-recognition), and more than half were methylation sensitive. For each enzyme, we performed *in silico* cuts of sequenced genomes of four plant species with sequenced genomes (*Arabidopsis thaliana*; *Oryza sativa*, rice; *Zea mays*, maize; *Glycine max*, soybean) (Table S2). This was done through the development and application of specific software, IgCoverage (File S1), to calculate the IgC as the total DNA fragments of different lengths (100–600 bp) as the ratio of the total fragment length over the sequenced genome of reported sequence length (as shown in Table S2). We considered a wider range of fragment lengths (100–600 bp) than the current GBS protocols (200–400 bp), mainly for relative IgC comparison and possible technical advances in future NGS platforms for GBS applications. The shell script IgCoverage1RE.sh in IgCoverage was run in a combination of 60 enzymes and four plant genomes. These IgC results (Table 1) were used to guide the development of new RE combinations for new two-enzyme GBS protocols as described below.

**Table 1:** The *in silico* genome coverages (IgC; %) of four plant species by DNA fragments of different lengths (100–600 bp) obtained from *in silico* digestions with 60 individual restriction enzymes

		*Arabidopsis*		Soybean		Rice		Maize			
Enzyme	SL	Count	IgC	Count	IgC	Count	IgC	Count	IgC	Mean	SD
*Cvi*AII	4	249,612	56.7	2,266,324	60.7	1,030,613	79.0	4,817,820	59.6	64.0	10.1
*Cvi*KI-1	4	376,984	67.3	2,648,781	61.9	1,150,523	58.9	6,059,914	53.9	60.5	5.6
*Nla*III	4	249,639	56.7	2,266,718	60.8	945,109	62.2	4,818,313	59.6	59.8	2.3
*Mlu*CI	4	360,972	64.7	2,790,706	57.9	969,836	56.2	4,546,748	54.1	58.2	4.6
*Cvi*RI	4	244,853	54.9	2,194,161	58.9	914,234	60.1	4,668,840	58.6	58.1	2.2
*Mse*I	4	349,553	65.9	2,583,144	58.7	821,137	51.2	3,442,333	43.4	54.8	9.7
*Alu*I	4	253,157	55.7	1,410,267	41.0	765,561	53.7	4,481,014	56.5	51.7	7.2
*Dpn*I	4	230,319	52.6	1,256,292	38.6	680,477	49.4	3,833,020	51.2	47.9	6.4
*Hinf*I	5	247,146	55.7	1,432,238	43.6	526,686	39.7	3,730,642	50.4	47.4	7.1
*Hpy*188I	5	224,589	51.4	1,181,936	35.8	574,679	42.6	3,713,268	48.5	44.6	6.9
*Dde*I	5	187,752	44.6	1,304,502	39.3	491,752	37.8	3,621,546	49.2	42.7	5.2
*Bfa*I	4	143,863	36.0	1,200,979	36.5	585,600	43.5	3,499,202	47.8	41.0	5.7
*Taq*I	4	172,656	41.6	638,273	19.9	510,683	37.5	3,315,820	44.3	35.8	11.0
*Hpy*CH4III	5	150,724	37.7	782,320	25.0	530,653	40.1	2,520,324	36.1	34.7	6.7
*Rsa*I	4	117,822	30.2	785,561	25.3	528,208	39.5	2,434,929	35.5	32.6	6.2
*Tfi*I	5	165,612	40.1	822,062	26.9	291,725	23.2	1,693,916	25.2	28.8	7.6
*Hae*III	4	41,199	11.0	498,808	15.1	464,330	33.0	3,238,709	40.6	24.9	14.2
*Fnu*4HI	5	65,979	16.8	368,604	11.5	479,530	32.9	2,810,234	35.0	24.1	11.6
*Hpy*CH4IV	4	107,461	27.1	544,678	16.9	342,538	26.3	1,565,649	22.7	23.3	4.6
*Sau*96I	5	39,880	10.8	483,707	15.0	355,928	26.9	2,817,258	37.3	22.5	12.0
*Scr*FI	5	38,190	10.0	289,281	9.2	350,643	25.9	2,680,165	34.3	19.9	12.3
*Msp*I	4	46,464	11.6	187,972	5.8	378,091	25.9	2,464,695	30.8	18.5	11.8
*Ape*KI	5	45,865	12.0	257,226	8.3	338,090	25.2	1,771,780	24.6	17.5	8.6
*Tsp*45I	5	50,386	13.5	385,719	12.7	200,604	16.6	1,249,590	18.8	15.4	2.8
*Hha*I	4	12,350	3.4	145,999	4.3	285,864	19.5	2,179,751	26.9	13.5	11.6
*Bst*NI	5	21,241	5.7	183,781	5.9	183,039	14.8	1,582,998	22.1	12.1	7.9
*Ava*II	5	22,727	6.3	241,494	7.8	144,992	11.8	1,395,284	20.0	11.5	6.2
*Acc*II	4	13,101	3.4	114,394	3.2	247,293	16.2	1,539,695	19.4	10.6	8.5
*Hpy*99I	5	19,497	4.8	55,184	1.6	207,880	13.8	1,401,069	18.2	9.6	7.7
*Ssp*I	6	44,863	11.4	493,702	15.5	92,294	7.3	213,496	3.3	9.4	5.3
*Bst*YI	6	41,760	11.1	189,122	6.3	105,736	8.7	621,055	9.9	9.0	2.0
*Nci*I	5	6272	1.7	55,716	1.9	125,439	9.4	973,572	13.3	6.5	5.7
*Psi*I	6	30,444	8.0	333,631	10.6	54,716	4.3	127,778	2.0	6.2	3.8
*Bsr*FI	6	5528	1.4	16,387	0.5	122,510	8.9	413,772	6.2	4.3	4.0
*Eco*T22I	6	9536	2.6	141,415	4.6	46,432	3.9	163,998	2.4	3.4	1.0
*Nsi*I	6	9536	2.6	141,415	4.6	46,432	3.9	163,998	2.4	3.4	1.0
*Hin*dIII	6	17,751	4.7	97,975	3.2	17,513	1.6	198,699	3.3	3.2	1.3
*BgI*II	6	8161	2.2	30,497	1.0	17,778	1.5	63,336	1.0	1.4	0.6
*Eco*RI	6	5555	1.6	45,234	1.5	11,684	1.0	65,745	1.1	1.3	0.3
*Ngo*MIV	6	166	0.0	1436	0.0	48,090	3.4	93,246	1.3	1.2	1.6
*Sac*I	6	2501	0.7	8413	0.3	20,000	1.6	141,985	2.1	1.2	0.8
*Xba*I	6	4329	1.2	36,162	1.3	12,426	1.1	69,100	1.1	1.2	0.1
*BgI*I	11	206	0.1	4433	0.2	27,650	2.1	152,478	2.3	1.2	1.2
*Pst*I	6	3047	0.9	13,079	0.4	16,785	1.5	85,561	1.4	1.0	0.5
*Eag*I	6	124	0.0	3898	0.1	32,765	2.5	100,460	1.4	1.0	1.2
*Afl*II	6	3637	1.0	36,561	1.3	5090	0.4	61,670	1.1	1.0	0.4
*Sph*I	6	853	0.2	21,649	0.8	18,406	1.6	67,646	1.1	0.9	0.5
*Alw*NI	9	2709	0.8	11,631	0.4	8836	0.8	95,178	1.6	0.9	0.5
*Bss*HII	6	12	0.0	1815	0.0	24,187	1.7	109,573	1.6	0.9	1.0
*Eco*RV	6	4015	1.1	19,768	0.7	9299	0.8	41,023	0.7	0.8	0.2
*Xho*I	6	1611	0.5	8640	0.3	8925	0.7	48,772	0.8	0.6	0.3
*Sac*II	6	182	0.0	862	0.0	15,281	1.1	60,642	0.9	0.5	0.6
*Bam*HI	6	1615	0.5	8977	0.3	6676	0.5	43,112	0.7	0.5	0.2
*Sal*I	6	530	0.1	5811	0.2	9303	0.7	53,675	0.7	0.5	0.3
*Sma*I	6	148	0.0	1524	0.1	6748	0.6	56,135	0.9	0.4	0.4
*Sgr*AI	8	96	0.0	390	0.0	14,315	1.1	23,916	0.3	0.4	0.5
*Kpn*I	6	429	0.1	7655	0.2	2887	0.3	28,522	0.5	0.3	0.1
*Not*I	8	0	0.0	5	0.0	992	0.1	2278	0.0	0.0	0.0
*Fse*I	8	3	0.0	59	0.0	749	0.1	1855	0.0	0.0	0.0
*Sbf*I	8	2	0.0	22	0.0	88	0.0	721	0.0	0.0	0.0

SL, site length; Count, the number of DNA fragments of different lengths (100–600 bp).

The software IgCoverage (File S1) was specifically developed and tested for *in silico* evaluation of expected genome coverage through GBS with an individual or a pair of REs on one sequenced genome. Two shell scripts, IgCoverage1RE.sh and IgCoverage2RE.sh, were written to calculate IgC values for an individual or a pair of REs, respectively. Both functions carry several major steps. First, the genome-sequence files in FASTA format of a species were downloaded from the NCBI database (Table S2). These FASTA files were renamed according to their respective chromosomes and used as input files. Second, the recognition sequence and cutting position of each RE was obtained as an input file and used to scan over a chromosome for the recognition site positions. Third, for each chromosome, DNA fragments were defined based on the positions of recognition sites. Four fragment metrics were recorded: the total number of DNA fragments, the total length of all DNA fragments, the number of DNA fragments of lengths 100–600 bp, and the total length of the DNA fragments within 100–600 bp. Fourth, a summation of the four fragment metrics over all the chromosomes was made for the whole genome. IgC was calculated for the species. The four fragment metrics and IgC value were saved as output. Fifth, the second to fourth steps were repeated for other REs, and an output file with four fragment metrics and IgC for each enzyme was generated. This script was rerun for each species.

### C2: development of new RE combinations

A total of 70 RE combinations (Table S3) were designed for *in silico* and empirical evaluations based on their genome coverage and/or SNP genotyping. These enzymes were paired or selected with the following considerations. First, we focused on two-enzyme GBS protocols with dual-indexing of sequencing runs, and pairs of enzymes were selected, as opposed to single enzymes. Second, enzymes needed to be readily available from a common supplier, capable of generating sticky (overhanging) ends for efficient ligation of adapters, and, preferably, to have compatible reaction buffers and the same incubation temperature to allow for simultaneous digestion. Third, we reasoned that the enzymes with shorter recognition sites would generate more, shorter fragments and that more fragments would lead to higher genome coverage. Thus, enzyme combinations with shorter recognition sites were favored and the following combinations were chosen: 40 5 + 4 bp, 25 6 + 4 bp, two 4 + 4 bp, and one 5 + 5 bp (Table S3). In addition, two RE combinations with longer recognition sites were selected: one 6 + 6 bp and one 6 + 8 bp (Table S3). For comparison, six RE combinations were also selected from previously published works (Vos *et al.* 1995; Maughan *et al.* 2009; Fu and Peterson 2012; Peterson *et al.* 2012; Poland *et al.* 2012a), including two long recognition site enzyme pairs, 6 + 6 bp and 8 + 6 bp. Fourth, plant genomes vary in overall GC content (Šmarda *et al.* 2014). Methylation of cytosine bases can also inhibit the ability of certain enzymes to cut DNA and could reduce the number of potential cut sites. Thus, some RE combinations were selected to address the variability in the GC content of the enzymes’ recognition sites (low, equal, and high, relative to the AT content). There were 47 enzyme combinations with at least one enzyme being methylation sensitive, as indicated in the supplier’s literature. Fifth, the enzymes were also favored if displaying high *in silico* IgC values in four model plant species (Table 1), and with even digestion patterns and/or large proportions of fragments less than 600 bp from *in vitro* tests over five plant species [rice, maize, soybean, wheat (*Triticum aestivum*), flax (*Linum usitatissiumum*)]. The *in vitro* digestions were conducted with 24 single REs and 23 RE combinations at 37° for 3 hr using 10 units of each enzyme and 100 ng of genomic DNA with the optimum buffer. Digests were separated on a 2% agarose gel at 100 V for 2 h and visualized by stain with GelRed (Biotium, Hayward, CA, USA) postrun.

### C3: in silico analysis of IgC for RE pairs

The *in silico* search for promising RE pairs had several steps. First, we downloaded 18 additional genome sequences from the NCBI database to enlarge the scope of the species assay (Table S2). Second, the shell script IgCoverage2RE.sh in IgCoverage (File S1) was applied to perform the *in silico* analysis of double-enzyme cutting of a sequenced genome for each RE combination. Specifically, IgCoverage2RE.sh simulated the digestion of the genome with two REs, and calculated the IgC value of the generated DNA fragments of different ends and with lengths 100–600 bp. Note that we considered only the DNA fragments of different ends and lengths within 100–600 bp as these fragments are likely sequenced in the current GBS protocols. The IgCoverage2RE.sh script was run for 70 RE combinations in one species and the run was repeated for the other 21 assayed species (Table S2). Third, we also assessed the distribution of the DNA fragments with different ends and lengths within 100–600 bp generated *in silico* by certain enzyme combinations on some plant genomes, and compared the abundance of DNA fragments generated by these RE combinations at particular genomic regions. Note that IgCoverage2RE.sh differs from IgCoverage1RE.sh mainly in that the former considers the cutting positions by two enzymes over a chromosome and only the DNA fragments of different ends for the IgC estimation.

### C4: empirical verification on plant species

The IgC verification was performed for three enzyme combinations (*Pst*I + *Msp*I = PM, *Ava*II + *Bfa*I = AB, and *Hin*fI + *Hpy*CH4IV = HH) on 12 plant species (Table 3). PM was the GBS reference RE pair and used for control, while AB and HH were selected to represent the enzyme combinations with the moderate and the high IgC values, respectively (see Table S3).

The HH pair was selected, as opposed to other RE pairs with higher IgC values, as it seemed to have the best combination of genome coverage, enzyme cost, and practical efficiency for verification on plant species. For example, *Hpy*CH4IV has a 50% GC-content recognition site (ACGT) compared to the AT-rich *Mse*I site (TTAA) and thus is more likely to avoid noncoding AT-rich regions (Haberer *et al.* 2005), especially in large, AT-rich genomes of cereals (Šmarda *et al.* 2014). The other RE pairs have different incubation temperatures (*e.g.*, *Cvi*AII at 25°, *Hin*fI and *Dde*I 37°, *Tfi*I 65°, and *Ape*KI 75°), requiring some modification of the current GBS protocols. Some of these pairs, such as *Mlu*CI/*Hin*fI, had higher IgC values for 22 species, but relatively lower IgC values in plant species. Also, *Bfa*I has a short shelf life and strict storage requirements, increasing its overall cost for a routine GBS use.

Six monocots (maize; rice; *Elymus lanceolatus*, northern wheatgrass; *Aegilops umbellulata*, goat-grass; *Pseudoroegneria spicata*, bluebunch wheatgrass; and *Agropyron cristatum*, crested wheatgrass) and six dicots (*Arabidopsis*; soybean; *Linum grandiflorum*, red flax; *Carthamus tinctorius*, safflower; *Pisum sativum*, pea; and *Sinapis alba*, yellow mustard) were selected to represent both model and nonmodel plant genomes of variable size. An individual plant with young leaf tissue collected from our previous genetic diversity research was selected to represent its species. DNA was extracted from leaf tissue and the same GBS procedures as described in Peterson *et al.* (2014) were used, but with adapters modified to anneal to the specific enzyme pairs. Two MiSeq runs were made, and each run consisted of three monocots and three dicots. The MiSeq Reporter software was set to produce demultiplexed data in both the forward and reverse directions in FASTQ format. The FASTQ files for each species/enzyme combination were processed separately.

The bioinformatics analysis of genome coverage for each enzyme combination in each species was performed in several steps. First, the MiSeq FASTQ data were cleaned to remove low quality sequences using Trimommatic (Bolger *et al.* 2014) with a five-base sliding window, and with a quality cut-off using a PHRED score of 24 and a minimum sequence length of 75 bases. Second, unique sequences were obtained independently from each enzyme and species combination using FastX_Collapser (Gordon 2010), followed by the *de novo* assembly of contigs using Minia (Salikhov *et al.* 2013). Minia was run with a k-mer size of 31, the minimum k-mer abundance of 2 and the genome size of 100,000,000. Third, the total number of contigs and the total number of bases from all contigs from each enzyme and species combination were counted with a custom Perl script. Empirical genome coverage (EgC) was estimated as the number of bases from all contigs against the estimated genome size of a plant species (listed in Table 3) from published flow cytometry values from the Plant DNA C-values Database, Kew Royal Botanical Gardens (http://data.kew.org/cvalues/). There was no report on the genome size of red flax, and its EgC was estimated using the reported genome size of cultivated flax in the Plant DNA C-values Database. Comparisons of EgC values for AB and HH to PM were also made for understanding genome sampling. Note that such a relative comparison of species-specific EgC or IgC values between two RE pairs should not be affected by the use of either estimated genome size (Table 3) or published genome sequence length (Table S2) of a species.

### C5: empirical verification on individual plants

The verification of IgC and SNP genotyping in 12 *Arabidopsis* and 12 rice individual samples was conducted with HH and PM. The assayed materials (Table 4) represented 12 races of *Arabidopsis* and 12 accessions of rice. Seeds were randomly selected from each race or accession and planted in the greenhouse at the Saskatoon Research and Development Centre. Young leaf tissue was specifically collected from a single plant representing a race or accession, and DNA was extracted. Following the gd-GBS protocol (Peterson *et al.* 2014), with adapters modified to anneal to the specific enzyme pairs, two additional MiSeq sequencing runs were conducted. Each MiSeq run consisted of all 48 indexed accession/enzyme combinations from both species, and generated 96 demultiplexed paired-end FASTQ files of each sample/enzyme combination for the two species. The contig assembly and SNP genotyping were performed using the npGeno pipeline with default settings (Peterson *et al.* 2014). The statistics on contigs and resulting SNPs, along with their read statistics, were compiled for each enzyme combination in each species. To assess the dynamics of missing values in SNP genotyping for each enzyme combination, total SNPs were analyzed and plotted with respect to the number of samples present and average number of reads per sample for both plant species.

### Data availability

Online supplementary information contains Figure S1, File S1, Table S1, Table S2, and Table S3. The genome sequence data for 22 assayed organisms are available in the NCBI database with direct links listed in Table S2. The original Illumina MiSeq data (in FASTQ format), with two runs for IgC verification on 12 plant species and with two runs for verification on 12 *Arabidopsis* and 12 rice samples, are available at the NCBI with Sequence Read Archive Project ID: SRP066269. The developed software, IgCoverage.rar (File S1), is available as an online supplementary file.

## Results

### Genome coverage from in silico analysis

The *in silico* digestions by 60 individual REs of the sequenced genomes of four plant species revealed a huge variation in the counts of DNA fragments of different lengths (100–600 bp) and a large variation in genome coverage by these generated DNA fragments in each species (Table 1). For example, the numbers of such DNA fragments obtained in *Arabidopsis* ranged from 0 (with the *Not*I enzyme) to 376,984 (with the *Cvi*KI-1 enzyme), and IgC values ranged from 0– 67.3% for these 60 enzymes. Such large variation in genome coverage was also observed for rice, maize, and soybean. The commonly used enzyme *Ape*KI displayed the average genome coverage of 17.5%, but there were 22 four- or five-cutter enzymes displaying more genome coverage than *Ape*KI in these four plant species (Table 1). Generally, frequent-cutter enzymes generated more DNA fragments and showed higher genome coverages. Overall, there were six four-cutter enzymes and 14 four-cutter or five-cutter enzymes having genomic coverages higher than 50% and 30%, respectively. The top cutter enzymes for these four species were either *Cvi*AII or *Cvi*KI-1, with the genome coverages larger than 60%.

The *in silico* digestions with 70 RE combinations of the sequenced genomes of 22 organisms also revealed a large variation in genome coverage in each species (Table 2 and Table S3). For example, the IgC values for 70 enzyme combinations ranged from 0.1–35.1% and averaged 14% in *Arabidopsis*, while they ranged from 0.1–30.1% and averaged 10.8% in fruit fly. For most of the RE pairs, there was also a large variation in IgC across the 22 species. Generally, larger IgC values were observed in plant than animal species. More variation was observed in the species with larger genomes. Overall, across the 22 species, there were 11 enzyme combinations having an average IgC of more than 20%, and 21 combinations with more than 15%. The top three RE pairs, with IgC values of 30% or higher across the 22 species, were *Cvi*AII + *Hin*fI, *Cvi*AII + *Dde*I, and *Bfa*I + *Hin*fI. In contrast, the commonly used enzyme combination *Pst*I + *Msp*I (or PM) displayed an averaged 4% IgC across the 22 species. The 21 enzyme combinations with the IgC values of 15% or higher (Table 2) are recommended for consideration for various GBS applications in diverse species, as they could generate 3.8–8 times more genome coverage than the GBS reference RE pair PM. These 21 RE pairs also displayed no significant associations detected between their IgC values and the genome sizes of the 22 species.

**Table 2:** The *in silico* genome coverages (IgC; %) of 22 species with sequenced genomes (eight plant, 13 animal, and one fungus) by DNA fragments of different ends and lengths (100–600 bp) obtained from *in silico* digestions with the top 21 restriction enzyme pairs and the GBS reference pair *Pst*I + *Msp*I

	Plant*^a^*								Animal													Fungi			
Enzyme Pair	Arabidopsis	Cottonwood	Medicago	Winegrape	Soybean	Rice	Sorghum	Maize	C. elegans	Fruit fly	Honey bee	Stickleback	Pike	Zebra fish	Turkey	Zebra finch	Dog	Housecat	House mouse	Pigmy chimp	Opossum	Baker’s yeast	Mean_all	SD_all	Mean_plants
Genome size (Mb)	120	379	297	426	950	382	659	2060	83	158	220	401	377	1340	1040	1021	2328	2419	2726	3152	2998	12			
*Cvi*AII + *Hin*fl	35.1	32.5	27.9	31.4	32.5	31.1	30.8	33.8	31.9	30.1	24.6	35.0	31.0	31.0	30.8	34.5	35.9	35.5	34.2	30.0	35.2	36.5	32.3	2.9	31.9
*Cvi*AII + *Dde*I	34.4	30.8	26.6	30.6	30.9	30.3	31.8	32.9	27.0	29.7	16.1	35.1	32.9	33.3	31.9	35.9	35.3	34.9	33.1	29.3	34.5	35.9	31.5	4.4	31.0
*Bfa*I + *Hin*fI	32.5	32.5	26.0	30.9	31.4	32.0	31.8	34.6	27.9	24.5	17.5	24.9	30.2	27.9	29.3	31.8	36.3	35.2	34.6	30.7	35.3	34.8	30.6	4.5	31.5
*Bfa*I + *Dde*I	32.5	31.4	25.0	30.4	30.4	31.2	32.3	34.2	23.8	24.0	11.8	25.1	30.8	29.1	28.7	30.9	33.6	32.9	32.4	29.0	33.6	34.4	29.4	5.1	30.9
*Cvi*AII + *Tfi*I	33.4	27.9	24.3	27.2	27.7	25.8	26.2	27.6	29.3	25.8	24.5	26.6	23.2	23.7	24.7	26.6	28.4	29.7	25.9	24.0	30.8	33.9	27.2	2.9	27.5
*Bfa*I + *Tfi*I	31.8	29.1	23.6	27.9	28.2	27.0	27.3	29.1	25.9	21.4	17.8	20.5	24.0	22.4	24.4	25.8	30.0	31.8	28.0	25.6	30.9	32.7	26.6	3.9	28.0
*Mlu*CI + *Hin*fI	25.3	18.0	14.1	19.8	19.2	24.9	28.1	29.1	13.5	20.7	14.1	33.0	28.4	24.2	25.1	26.6	29.6	29.4	30.1	23.5	24.5	24.2	23.9	5.5	22.3
*Mlu*CI + *Dde*I	24.4	17.3	13.6	19.4	18.6	24.0	27.5	29.0	10.2	19.6	7.4	33.3	30.1	26.0	26.5	28.4	28.6	29.4	29.2	23.5	24.1	22.3	23.3	6.7	21.7
*Cvi*AII + *Ape*KI	19.3	17.2	12.9	13.4	14.2	25.3	22.6	23.8	18.5	27.7	11.0	33.7	25.6	26.6	28.1	31.8	24.9	23.9	25.9	21.0	20.9	25.4	22.4	6.0	18.6
*Hin*fI + *Mse*I	28.1	22.0	18.7	24.7	24.3	28.0	28.8	30.6	27.0	7.9	4.3	10.4	9.8	21.6	8.4	28.3	28.8	28.7	11.2	26.1	7.0	31.6	20.7	9.2	25.6
*Hin*fI + *Hpy*CH4IV	29.4	22.7	21.6	18.0	23.1	27.9	27.2	27.9	30.5	14.3	11.6	16.1	14.8	24.8	11.7	16.0	19.4	22.7	9.3	15.8	5.6	35.4	20.3	7.7	24.7
*Dde*I + *Mse*I	27.1	20.8	17.9	24.1	23.2	26.9	29.3	30.3	21.5	8.3	3.7	10.2	9.3	22.6	6.0	29.5	29.0	29.1	7.5	25.1	5.5	30.0	19.9	9.5	25.0
*Mlu*CI + *Tfi*I	23.7	15.0	11.8	16.6	15.9	19.9	23.3	24.6	11.8	16.9	13.1	24.8	21.0	18.4	19.6	19.8	22.7	25.3	22.6	18.5	20.4	20.6	19.4	4.1	18.9
*Dde*I + *Hpy*CH4IV	29.2	22.2	20.8	17.5	22.6	27.2	27.2	27.5	26.3	14.9	9.3	15.9	13.8	26.0	8.6	14.6	18.0	20.7	6.5	14.2	4.7	35.2	19.2	8.1	24.3
*Ape*KI + *Bfa*I	19.5	18.3	13.1	14.9	15.1	26.3	23.3	24.3	16.8	12.6	5.9	13.8	14.7	25.2	13.5	29.8	26.6	23.9	14.1	22.8	10.5	24.3	18.6	6.3	19.4
*Tfi*I + *Hpy*CH4IV	29.0	20.7	19.7	16.8	21.5	24.2	24.2	23.7	28.3	13.9	12.3	15.2	13.2	20.2	10.9	13.3	16.8	20.7	9.1	13.8	6.1	33.3	18.5	6.9	22.5
*Nla*III + *Mlu*CI	25.7	19.4	16.0	20.2	19.6	25.4	27.0	29.0	13.4	5.2	2.4	10.8	7.7	27.1	6.3	28.7	29.3	30.2	8.0	24.3	5.4	24.3	18.4	9.4	22.8
*Tfi*I + *Mse*I	26.5	19.0	16.3	21.5	20.9	23.6	24.3	26.3	24.5	7.8	5.0	9.7	8.5	16.0	7.7	21.4	22.2	23.9	10.1	21.0	7.1	28.3	17.8	7.5	22.3
*Cvi*AII + *Ava*II	15.6	14.0	12.3	14.5	14.6	18.7	20.8	24.7	12.4	15.9	7.0	22.9	19.6	13.4	14.5	17.7	20.4	20.9	22.3	14.9	20.0	18.1	17.1	4.2	16.9
*Ape*KI + *Mse*I	17.2	14.1	9.9	13.2	12.8	25.1	22.9	23.2	15.8	7.2	3.8	10.0	9.5	19.5	7.0	26.7	21.8	20.4	10.8	19.2	6.2	21.3	15.3	6.8	17.3
*Ava*II + *Bfa*I	16.1	15.3	12.3	15.8	15.5	20.6	21.8	26.2	11.3	9.0	3.9	11.6	12.3	13.0	9.0	17.3	22.5	21.4	13.4	16.2	10.0	18.1	15.1	5.3	17.9
*Pst*I + *Msp*I	3.5	2.3	1.6	1.8	1.5	5.6	6.0	5.5	3.1	4.5	0.6	8.9	4.8	6.2	3.3	5.3	5.4	4.6	3.0	5.0	0.8	4.4	4.0	2.0	3.5

IgC values for additional 48 restriction enzyme pairs are shown in Table S3. Three enzyme pairs selected for empirical evaluations in plants are highlighted in red. The number below the organism is its genome size in Mbp (1 × 10^6^ bp). More genome information for these species is shown in Table S2.

a The organisms listed are the same as Table S2.

**Figure 2:**
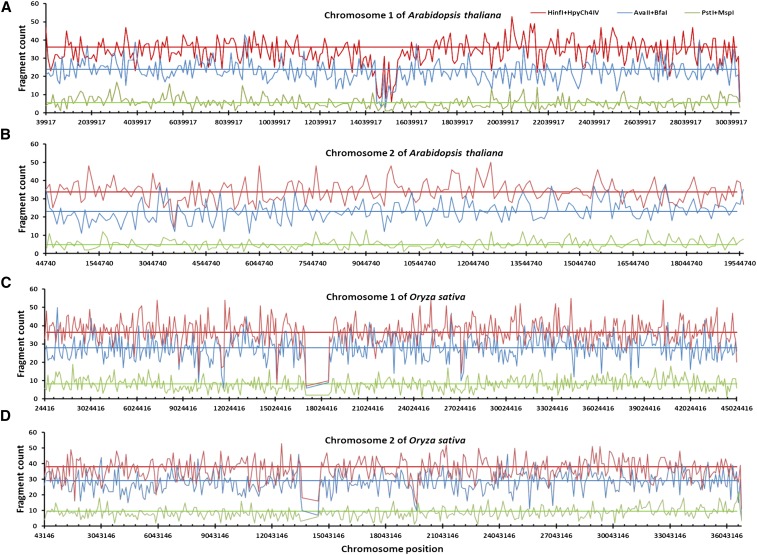
Fragment distributions detected in *silico* on selected chromosomes of *Arabidopsis* and rice. Distributions of DNA fragments generated by *in silico* digestions with three restriction enzyme combinations on two chromosomes of *Arabidopsis thaliana* (A, B) and *Oryza sativa* (C, D). The number of DNA fragments and the average enzyme-cutting position based on a 100 kb sliding-window of a given chromosome are calculated and shown with a colored line for each enzyme combination. The corresponding horizontal linear line represents the average fragment count for an enzyme combination on the chromosome. More digestions were found for *Hin*fI + *Hpy*Ch4IV than the other two enzyme combinations.

To verify the differences in genome coverage among these enzyme combinations, we also assessed DNA fragment distributions for the *in silico* digestions by three selected RE combinations (PM, AB, HH) on all the chromosomes of *Arabidopsis* and rice. Figure 2 shows the DNA fragment distributions on two chromosomes of *Arabidopsis* and rice. Clearly, HH generated more different DNA fragments than AB and PM on the assayed chromosomes of each species. For example, HH had an average of 37 DNA fragments on each 100 kb sliding-window of chromosome 1 of *Arabidopsis*, while AB and PM had only 23 and 7, respectively. Similarly, HH had an average of 36 DNA fragments on each 100 kb sliding-window of chromosome 1 of rice, while AB and PM had only 28 and 8, respectively. Note that the striking drops in fragment counts (Figure 2, C and D) reflected the assembly gaps in the respective rice chromosomes.

### Genome coverage at the species level

We performed an empirical verification of genome coverage for the three selected RE combinations (PM, AB, HH) with two runs of MiSeq GBS sequencing on six monocot and six dicot plant species. Several interesting results emerged (Table 3). First, the numbers of contigs and the averaged contig lengths generated by each enzyme combination varied substantially, with variable genome sizes of the 12 species. Second, each enzyme combination also displayed a wide range of variation in genome coverage across the 12 species. For example, PM displayed EgC values ranging from 0.1–3.02%, and HH had EgC values ranging from 0.55–7.90%. Third, larger EgC values were observed for AB and HH than for PM in each species. For example, the average EgC across these 12 species was 0.88% for PM, 2.28% for AB, and 2.85% for HH. The same pattern of variation was observed when either monocots or dicots were considered separately. Fourth, AB and HH displayed 3.27 and 3.45 times more genome coverage than PM across the 12 species, respectively. Interestingly, AB and HH displayed larger EgC values in dicots than in monocots. Fifth, the linear regression analyses revealed significant associations of the EgC values obtained by AB and HH, but not by PM, with the genome sizes of the 12 assayed plant species (Figure S1). Smaller EgC values for AB and HH were observed in species of larger genome size.

**Table 3:** The empirical genomic coverages (EgC) for three restriction enzyme combinations (PM = *Pst*I + *Msp*I, AB = *Ava*II + *Bfa*I, and HH = *Hin*fI + *Hpy*CH4IV) and the ratio of the EgC relative to PM (Ratio to PM) in six dicot and six monocot species

		PM		AB			HH		
Plant Species	GS*^a^*	Contig × Length*^a^*	EgC (%)	Contig × Length	EgC (%)	Ratio to PM	Contig × Length	EgC (%)	Ratio to PM
Dicot									
* Arabidopsis thaliana*	156	17,352 × 208	0.74	51,231 × 218	2.28	3.09	111,175 × 196	4.45	6.03
* Pisum sativum*	4768	27,703 × 175	0.10	355,620 × 127	0.95	9.35	203,041 × 130	0.55	5.45
* Linum grandiflorum*	685	53,747 × 173	1.36	122,437 × 142	2.55	1.87	202,992 × 147	4.37	3.21
* Carthamus tinctorius*	1364	22,686 × 194	0.32	166,661 × 150	1.83	5.67	159,252 × 148	1.72	5.34
* Glycine max*	1100	45,759 × 171	0.71	222,148 × 165	3.33	4.67	224,215 × 163	3.33	4.67
* Sinapis alba*	489	39,423 × 191	1.54	141,589 × 167	4.83	3.13	182,384 × 172	6.41	4.16
Mean			0.80		2.63	4.63		3.47	4.81
Monocot									
* Aegilops umbellulata*	4939	128,544 × 164	0.43	294,115 × 126	0.75	1.76	311,323 × 148	0.93	2.19
* Pseudoroegneria spicata*	4450	186,910 × 159	0.67	466,001 × 129	1.35	2.02	365,274 × 151	1.24	1.85
* Agropyron cristatum*	6969	264,949 × 147	0.56	549,794 × 126	1.00	1.78	439,060 × 147	0.92	1.65
* Zea mays*	2665	84,632 × 182	0.58	295,130 × 136	1.51	2.62	293,728 × 147	1.62	2.81
* Elymus lanceolatus*	8240	275,761 × 157	0.52	368,636 × 131	0.59	1.12	416,130 × 145	0.73	1.39
* Oryza sativa*	489	75,742 × 195	3.02	164,171 × 190	6.36	2.11	209,403 × 184	7.90	2.61
Mean			0.96		1.93	1.90		2.22	2.08
Overall mean			0.88		2.28	3.27		2.85	3.45

a GS, genome size in Mb obtained from Royal Botanic Gardens, Kew Plant DNA C-values Database; GS is not available for *L. grandiflorum*, so GS for the related flax species (*L. usitatissiumum*) was used. Contig × length = number of contigs × average length (bp) per contig.

### Genome coverage and SNP discovery at the individual level

We also performed an empirical verification of genome coverage for two selected RE combinations (PM, HH) with two runs of Miseq GBS sequencing on 12 *Arabidopsis* and 12 rice plants and found several patterns of variation (Table 4). First, the numbers of contigs and the averaged contig lengths generated by each enzyme combination varied greatly among the 12 samples of each species. Second, each enzyme combination displayed more contigs (on average) in the rice than *Arabidopsis* samples. Third, HH displayed 2.8 and 2.6 times more genome coverage (average of all the samples) than PM in *Arabidopsis* and rice samples, respectively. For the sample-wise genome coverage, this pattern was also true, but more variation in sample-wise genome coverage was observed in *Arabidopsis* than rice samples.

**Table 4:** Statistics of contig and mean contig length per sample obtained for two restriction enzyme combinations (PM = *Pst*I + *Msp*I; HH = *Hin*fI + *Hpy*CH4IV) in combined runs of 12 *Arabidopsis* and 12 rice samples

	PM		HH		
Sample	Contig Count	Mean Length	Contig Count	Mean Length	Length Ratio*^a^*
*Arabidopsis*					
Bur-0	59,354	204	120,602	208	2.1
Col-0	79,750	199	154,377	206	2.0
Col-1	25,865	198	89,198	215	3.8
Col-2	24,647	201	84,160	217	3.7
Col-3	25,891	200	109,742	218	4.6
Col-4	62,244	202	134,119	212	2.3
Col-5	27,325	201	99,377	223	4.0
Col-6	35,066	200	105,837	216	3.3
Col-7	38,725	203	95,451	219	2.7
LER	73,058	197	131,169	213	1.9
Tsu-1	75,930	198	123,256	210	1.7
WS4	63,640	199	113,102	214	1.9
Mean	49,291	200	113,366	214	2.8
Rice					
R163	66,984	220	176,323	213	2.5
R237	63,211	223	206,916	214	3.1
R242	62,994	221	220,699	209	3.3
R286	64,506	221	158,090	210	2.3
R423	61,157	219	181,826	212	2.9
R614	70,942	213	176,441	210	2.4
R735	56,315	209	140,580	205	2.4
R971	73,663	221	199,219	211	2.6
R1120	63,652	220	146,569	211	2.2
R1409	58,519	220	170,706	209	2.8
R1570	69,318	218	166,983	211	2.3
R1662*^b^*	5690	246	164,098	214	25.0
Mean	64,660	219	176,759	210	2.6

a Length ratio, the ratio of the total base pairs obtained for HH over those for PM.

b Rice sample R1662 had a sequencing issue with PM and was excluded from the mean calculations.

We also conducted SNP genotyping for each of the two selected RE combinations in each species using the npGeno pipeline, and generated some basic statistics of contigs and SNPs obtained from the GBS SNP discovery with respect to sequence read and missing data (Table 5). First, more contigs with fewer sequence reads were obtained for HH than PM for either species, suggesting more genome coverage for HH. Accordingly, more average reads per contig were observed for PM than HH. For example, the average read per contig per sample for PM was 50.3 in *Arabidopsis* and 16.1 in rice, while for HH they were 10.7 in *Arabidopsis* and 8.0 in rice. Second, more SNPs with fewer reads per SNP per sample were identified for HH than PM for either species. For example, HH displayed 11,489 and 11,526 SNPs in *Arabidopsis* and rice, respectively, while PM showed 1343 and 7886 SNPs in *Arabidopsis* and rice, respectively.

**Table 5:** Statistics of contigs and single nucleotide polymorphisms obtained for two restriction enzyme combinations (PM = *Pst*I + *Msp*I; HH = *Hin*fI + *Hpy*CH4IV) in combined runs of 12 *Arabidopsis* and 12 rice samples

	*Arabidopsis*		Rice	
Statistic	PM	HH	PM	HH
Contig statistic				
Total contigs	10,498	42,355	28,611	36,808
Mean contig length (SD) in bp	243 (18)	242 (20)	239 (18)	238 (19)
Total reads	6,334,545	5,431,330	5,514,707	3,547,509
Mean reads/contig	603.4	128.2	192.7	96.4
Mean reads/contig/sample	50.3	10.7	16.1	8.0
Contigs with SNP0 (%)	239 (2.3)	473 (1.1)	1308 (4.6)	852 (2.3)
Contigs with SNP0 + SNP1 (%)	368 (3.5)	1623 (3.8)	1960 (6.9)	1900 (5.2)
Contigs with SNP0 + SNP1 + SNP2 (%)	453 (4.3)	2915 (6.9)	2319 (8.1)	2834 (7.7)
Contigs with SNPwt (%)	672 (6.4)	5405 (12.8)	3687 (12.9)	5096 (13.8)
SNP statistic				
Total SNPs	1343	11,489	7886	11,526
Total reads	350,269	925,468	1,412,233	1,052,072
Mean reads/SNP	260.8	80.6	179.1	91.3
Mean reads/SNP/sample	21.7	6.7	14.9	7.6
SNP0 (%)	423 (31.5)	1122 (9.8)	2325 (29.5)	1995 (17.3)
SNP0 + SNP1 (%)	688 (51.2)	3417 (29.7)	3823 (48.5)	4216 (36.6)
SNP0 + SNP1 + SNP2 (%)	884 (65.8)	6168 (53.7)	4753 (60.3)	6261 (54.3)

The percent of the total contigs or total SNPs (single nucleotide polymorphisms) is shown in parenthesis. SNP0, SNPs having no missing observations across the 12 samples; SNP1, SNPs having 8.3% observations missing (or absent in one of the 12 samples); SNP2, SNPs having 16.7% observations missing (or absent in two of the 12 samples); SNPwt, SNPs with or without missing observations across the 12 samples.

To understand if a new enzyme combination can also improve GBS SNP genotyping with more SNPs having fewer missing observations, we performed further SNP analysis to assess the extent of missing data in SNP genotyping for each selected RE combination in either species. It was found that there were many more SNPs with 0–16.7% missing observations [or absent in zero, one, or two (out of 12) samples] for HH than PM in either species (Table 5). For example, there were 6168 SNPs for HH *vs.* 884 SNPs for PM in *Arabidopsis*, and 6261 SNPs for HH *vs.* 4753 SNPs for PM in rice. Also, there were more contigs detected (with SNPs having up to 16.7% observations missing) for HH than for PM in either species. For example, there were 5405 contigs for HH *vs.* 672 contigs for PM in *Arabidopsis*, and 5096 contigs for HH *vs.* 3687 contigs for PM in rice.

**Figure 3:**
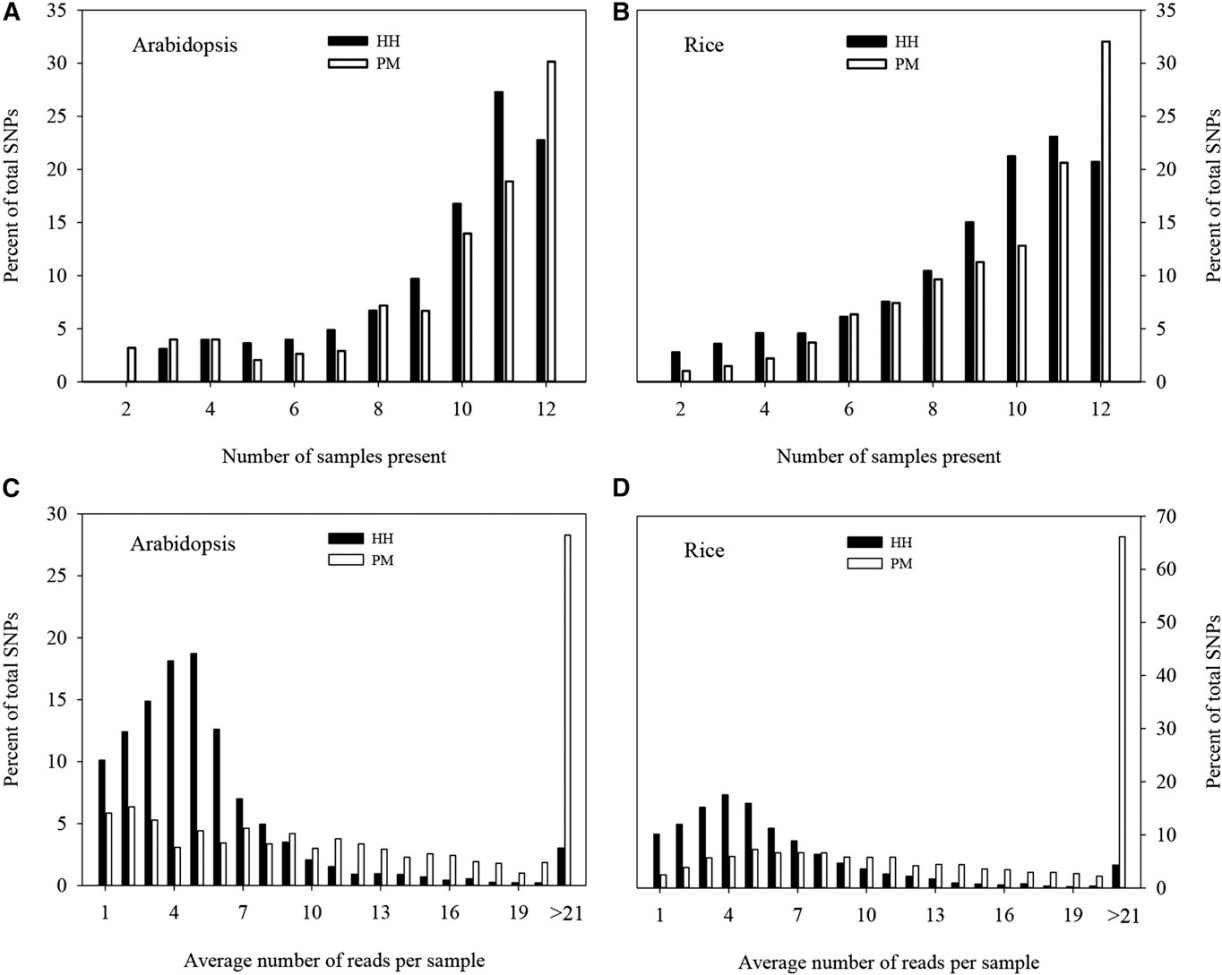
SNP distributions for two RE pairs in *Arabidopsis* and rice. Distribution of total single nucleotide polymorphisms detected with respect to the number of samples present, and average number of reads per sample, for two restriction enzyme combinations (PM = *Pst*I + *Msp*I; HH = *Hin*fI + *Hpy*CH4IV) in combined runs of 12 *Arabidopsis* and 12 rice samples.

To understand the dynamics of missing data in SNP genotyping under a new enzyme combination, we assessed the distribution of total SNPs detected with respect to read count per sample and to the number of samples present (or those having the SNP) for two enzyme combinations (PM, HH) in *Arabidopsis* and rice samples (Figure 3). It is clear that PM displayed more SNPs without missing observations (or present in all 12 samples) than HH in either species (Figure 3, A and B). This is not surprising, as SNPs from PM had more reads per sample in either species (Figure 3, C and D). For example, 28% (*Arabidopsis*) and 66% (rice) of the detected SNPs for PM had more than 21 reads per sample, while the majority of the detected SNPs from HH had only 4–7 reads per sample. However, it was also observed that the combined number of SNPs with 0–16.7% missing observations was larger for HH than PM in either species (Figure 3, A and B).

## Discussion

Our search for better RE combinations has produced not only a useful *in silico* analytical tool, IgCoverage (File S1), for further analysis of an individual or a pair of REs for GBS applications in a species of particular interest, but also revealed a novel set of 21 combinations of four- or five-cutter REs with significant increases in genome coverage for different GBS applications (Table 2). The empirical evaluation of the *Hin*fI + *Hpy*CH4IV combination in 12 plant species yielded 1.7–6 times more genome coverage than the commonly used RE pair *Pst*I + *Msp*I, and 2.3 times more genome coverage in dicots than monocots (Table 3). Further SNP genotyping analysis showed that *Hin*fI + *Hpy*CH4IV generated 7 and 1.3 times more SNPs with 0–16.7% missing observations than *Pst*I + *Msp*I in separate MiSeq sequencing runs of 12 *Arabidopsis* and 12 rice plants, respectively (Table 5). These findings demonstrated the potential of new enzyme combinations for advancing GBS applications with increased genome sampling and improved SNP genotyping.

Our search focused on the two-enzyme system, but the *in silico* analysis of genome coverage for a single enzyme system in four plant species is also encouraging. For example, the four-cutter enzyme *Cvi*AII showed an averaged genome coverage of 64%, while the commonly used enzyme *Ape*KI displayed an averaged genome coverage of 17.5%. There were 22 four- or five-cutter enzymes displaying more genome coverage than *Ape*KI in these plant species (Table 1). Thus, it is possible for further improvement of GBS protocols with the selection and evaluation of the single enzyme system of genome reduction. More research is needed in this direction to search for better genome sampling with adequate read depth (Elshire *et al.* 2011; Beissinger *et al.* 2013; Schilling *et al.* 2014).

The *in silico* analysis of the two-enzyme system was not exhaustive in either enzyme combination or assayed organism, but our search scope was much larger than those reported so far (*e.g.*, Poland *et al.* 2012a; Peterson *et al.* 2012; Heffelfinger *et al.* 2014). In spite of this, searching further for better enzyme combinations for increased genome coverage is still encouraged, particularly for a specific species of interest. Also, we focused only on the genome coverage and did not consider the issue of read depth, as the latter can be optimized with a given sequencing effort (Beissinger *et al.* 2013). However, our effort generated an encouraging outcome with a novel set of 21 combinations of four- or five-cutter REs with increased genome coverage when compared with the GBS reference pair *Pst*I + *Msp*I. The enzyme combinations with large genome coverages appeared to be those four- or five-cutter pairs (Table 2). There was only one pair of five-cutters (*Ava*II + *Ape*KI) displaying an IgC value of 22.4%, and one pair of four-cutters (*Msp*I + *Mse*I) with a value of 11.7%, across the 22 species. These results suggest that the search for the best two-enzyme GBS protocol should focus on those combinations of four- or five-cutters. Also, the increases in genome coverage for these enzyme combinations vary greatly across the 22 assayed species. This variation may reflect the genome size and structure. For example, the plant species with larger genomes (or abundant repeat sequences and genome duplication) appeared to show higher genome coverages (Table 2). Such variation may also reflect the selection of DNA fragments with different ends and lengths within 100–600 bp, as the other DNA fragments not matching these criteria were excluded from consideration for genome coverage.

One encouraging finding in empirical verifications in plants is that the enzyme combinations with larger EgC values also generated many more SNPs with a mild (0–16.7%) level of missing data than *Pst*I + *Msp*I. Also, it was found that the read depths of such SNPs are more shallow (with 4–7 reads on average) in these enzyme combinations, suggesting error rates for these SNPs would be expected to be higher than those using *Pst*I + *Msp*I. We focused more on the counts of GBS SNPs with the mild level of missing observations, and less on the accuracy of the SNPs and the optimization of read depth *vs.* SNP density. All of these issues are dependent on the total sequencing output of a GBS effort (Wendl 2006; Sims *et al.* 2014). Higher accuracy of SNPs requires an increased sequence read output per sample, through either more sequencing runs with higher sequencing cost or decreased multiplexing with fewer samples. More research is needed to optimize the parameters of genome coverage, read depth, SNP accuracy, and sequencing effort for a defined research goal in a species (Beissinger *et al.* 2013; Heffelfinger *et al.* 2014). This optimization is critical for the application of GBS to species with large genomes. *e.g.*, grass species. The expected number of DNA fragments generated by an enzyme combination is proportional to the genome size. The more DNA fragments generated for larger genomes, the more sequencing output is needed to identify accurately the fragment with adequate read depth (Beissinger *et al.* 2013; Sims *et al.* 2014).

The empirical verification also revealed smaller values of genome coverage for those assayed enzyme combinations than those obtained from the *in silico* analysis (Table 2 and Table 3). Such variation should not be surprising for several reasons. First, many factors may have contributed to the selection and identification of the DNA fragments. The *in silico* analysis considered much wider fragment length (100–600 bp), while the actual GBS runs may consider only those of length 200–400 bp. Bioinformatics analysis may have also presented some bias in excluding some DNA fragments for contig identification for both comparative enzyme combinations. It is also possible that some contigs generated by Minia may not be fully unique and still had overlapping sequences among some contigs, biasing upward the genome coverage calculation. Second, a sequencing flow cell has a finite number of binding sites for fragments and, once saturated, additional fragments cannot be sequenced. This technical feature would greatly affect the efficiency of more frequently cutting RE pairs in read output. This was evident as more frequently cutting RE pairs had a lower read depth compared to less frequently cutting combinations (Table 5). Third, our verification effort focused more on the comparative genome coverage, but not on the absolute extent of gain in genome coverage. It was our goal to explore those candidate RE combinations with possible gain in genome coverage, not to test and develop the best RE combination in a specific species.

Our empirical verifications were performed on plant species with a few selected enzyme combinations to illustrate the potential of improvement in genome coverage, thus biasing against animal species. The *in silico* analysis revealed 21 enzyme combinations with 15% or higher genome coverage in a wide range of plant, animal, and fungus species, which is much more than *Pst*I + *Msp*I (4%). More research is needed to verify some of these enzyme combinations in animal species. This is especially important, given the finding of larger variation in genome coverage in 13 animal species (Table 2 and Table S3). Similarly, our *in silico* analysis of the single enzyme system was done only with plant species. It is possible to expand the search effort for animal species, along with empirical verification (De Donato *et al.* 2013). Our search for higher genome coverage and the developed associated software were largely aimed at genetic diversity and population genetic studies, but both are equally useful for research into different interests focusing on a few hundred loci with higher read depth. For example, searching with IgCoverage for REs with less genome coverage would allow for an optimized experimental design with more samples to be assayed in a sequencing lane.

We are confident, however, that these novel enzyme combinations (Table 2) will play a role in future GBS applications, as these RE combinations can increase genome sampling and improve SNP genotyping. For optimum use of these RE combinations in GBS protocols, several factors are worth mentioning for consideration. First, when selecting an enzyme combination based on its performance in this study, it should be used with a species closely related to one of the relevant species assayed *in silico* here. Alternatively, a new *in silico* analysis using IgCoverage should be pursued to assess specific REs or pairs of REs in the genome sequence of the species of interest, or that of a closely related species if sequence data are not available for the first. Second, the adapters used in the GBS protocol need to be modified to anneal to the fragment ends generated by the selected enzyme combination. Third, a preliminary empirical assessment of genome coverage for the selected RE or RE combination in the species of interest is recommended before pursuing a large scale GBS application. Fourth, some effort may also be needed to optimize the enzyme combination for use in a specific species with respect to sequence output, read depth, SNP accuracy, and the extent of multiplexing (Hamblin and Rabbi 2014; Heffelfinger *et al.* 2014). Pursuing too high a degree of genome coverage may not always be the best option, as it may generate less accurate SNPs with a given sequencing effort (Beissinger *et al.* 2013). Fifth, some attention should be paid to the limitations of our search effort, the desired level of genome coverage and the workable level of missing observations in SNP data.

### Conclusion

Our *in silico* analyses of 22 plant, animal, and fungus species produced a novel set of 21 combinations of four- or five-cutter REs with increased genome coverage for a GBS application. The empirical evaluations of some new enzyme combinations in plant species confirmed the increase in genome coverage and the improvement in SNP genotyping. The developed *in silico* analytical tool, IgCoverage, should also be useful for further analysis of an individual or a pair of REs for GBS applications in a species of particular interest.

## Supplementary Material

Supporting Information
