# Supplementary material for: Increasing Genome Sampling and Improving SNP Genotyping for Genotyping-by-Sequencing with New Combinations of Restriction Enzymes
Source: G3 (Bethesda). 2016 Jan 27;6(4):845–56. doi: 10.1534/g3.115.025775 (PMC4825655; doi:10.1534/g3.115.025775)
Supplement: Supporting Information [file supp_6_4_845__index.html]

Increasing Genome Sampling and Improving SNP Genotyping for Genotyping-by-Sequencing with new Combinations of Restriction Enzymes — Increasing Genome Sampling and Improving SNP Genotyping for Genotyping-by-Sequencing with New Combinations of Restriction Enzymes — Supporting Information 

# Increasing Genome Sampling and Improving SNP Genotyping for Genotyping-by-Sequencing with New Combinations of Restriction Enzymes

## Supporting Information for Fu, Peterson, and Dong, 2016

**Files in this Data Supplement:**

- Figure S1 - The empirical genome coverages (%) obtained for three restriction enzyme combinations (PM=PstI+MspI; AB=AvaII+BfaI; HH=HinfI+HpyCH4IV) in 12 plant species in relations to their genome sizes. (.pdf, 13 KB)
- Table S1 - List of 60 restriction enzymes used in this study, along with their catalogue numbers and basic features: recognition site and length, reported methylation sensitivity, active temperature (Temp) and unit cost (¢/unit). (.pdf, 108 KB)
- Table S2 - List of 22 species (8 plant, 13 animal and 1 fungus) and their genome sequence information obtained from NCBI database for *in silico* analysis of restriction enzyme digestions. (.pdf, 24 KB)
- Table S3 - The *in silico* genome coverages (IgC; %) of 22 species with sequenced genomes (8 plant, 13 animals and 1 fungus) by DNA fragments of different ends and lengths (100-600bp) obtained from *in silico* digestions by 70 restriction enzyme pairs, including the GBS reference pair PstI+MspI. (.pdf, 117 KB)
- File S1 - Supplemental file for the software *IgCoverage.rar* and "Getting Started with IgCoverage.pdf." (.rar, 320 KB)
