# Supplementary material for: Increasing Genome Sampling and Improving SNP Genotyping for Genotyping-by-Sequencing with New Combinations of Restriction Enzymes
Source: G3 (Bethesda). 2016 Jan 27;6(4):845–56. doi: 10.1534/g3.115.025775 (PMC4825655; doi:10.1534/g3.115.025775)
Supplement: Supporting Information [file supp_g3.115.025775_FigureS1.pdf]

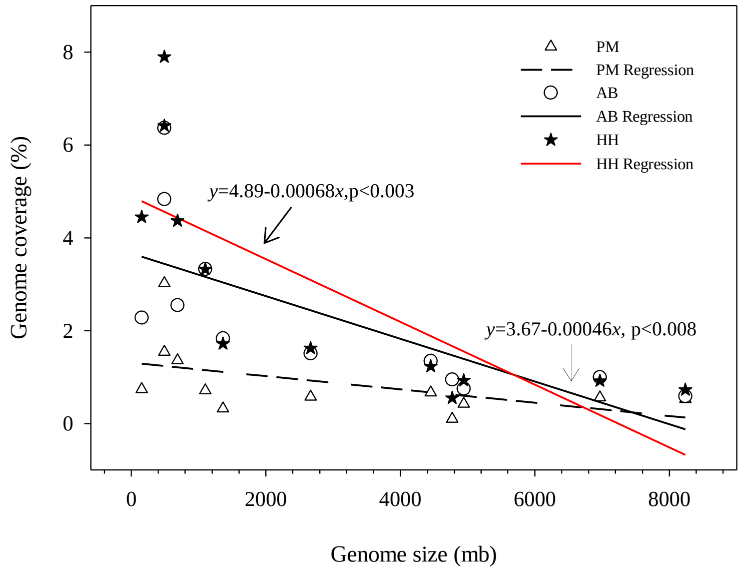

Figure S1 The empirical genome coverages (%) obtained for three restriction enzyme combinations (PM=PstI+MspI; AB=AvaII+BfaI; HH=HinfI+HpyCH4IV) in 12 plant species in relations to their genome sizes. Significant linear regressions (in solid line) were detected for AB and HH
