# Supplementary material for: Increasing Genome Sampling and Improving SNP Genotyping for Genotyping-by-Sequencing with New Combinations of Restriction Enzymes
Source: G3 (Bethesda). 2016 Jan 27;6(4):845–56. doi: 10.1534/g3.115.025775 (PMC4825655; doi:10.1534/g3.115.025775)
Supplement: Supporting Information [file supp_g3.115.025775_TableS1.pdf]

Table S1 List of 60 restriction enzymes used in this study, along with their catalogue numbers and basic features: recognition site and length, reported methylation sensitivity, active temperature (Temp) and unit cost (¢/unit)

Enzyme [a]	Catalogue number	Recognition site	Site length	Methylation	Temp (°C)	¢/unit
AccII (FnuDII)	Clontech 1002A	CG|CG	4	CpG	37	45
AflII	R0520L	C|TTAAG	6	No	37	3.4
AluI	R0137L	AG|CT	4	No	37	7.2
AlwNI	R0514L	CAGNNN|CTG	9	dcm	37	14.4
ApeKI	R0643L	G|CWGC	5	CpG	75	29.8
AvaII	R0153L	G|GWCC	5	CpG	37	3.4
BamHI	R0136L	G|GATCC	6	No	37	0.6
BfaI	R0568L	C|TAG	4	No	37	15.6
BglI	R0143L	GCCNNNN|NGGC	11	CpG	37	3.2
BglII	R0144L	A|GATCT	6	No	37	3.2
BsrFI	R0562L	R|CCGGY	6	CpG	37	6.9
BssHII	R0199L	G|CGCGC	6	CpG	37	14.4
BstNI	R0168L	CC|WGG	5	No	60	2.1
BstYI	R0523L	R|GATCY	6	No	60	3.4
CviAII	R0640L	C|ATG	4	No	25	34.3
CviKI-1 (CviTI)	R0710L	RG|CY	4	No	37	29.8
CviRI	Jin *et al.* 1994	TG|CA	4	CpG	23	n/a
DdeI	R0175L	C|TNAG	5	No	37	7.2
DpnI	R0176L	GA|TC	4	CpG	37	6.9
EagI	R3505L	C|GGCCG	6	CpG	37	13.7
EcoRI	R3101L	G|AATTC	6	CpG	37	0.6
EcoRV	R0195L	GAT|ATC	6	CpG	37	1.6
EcoT22I (AvaIII)	Clontech 1125A	ATGCA| T	6	No	37	3.8
Fnu4HI	R0178L	GC|NGC	5	CpG	37	36.1
FseI	R0588L	GGCCGG|CC	8	CpG,dcm	37	77.8
HaeIII	R0108L	GG|CC	4	No	37	2.2
HhaI	R0139L	GCG|C	4	CpG	37	3.2
HindIII	R3104L	A|AGCTT	6	No	37	0.6
HinfI	R0155L	G|ANTC	5	CpG	37	1.3
Hpy188I	R0617L	TCN|GA	5	dam	37	7.2
Hpy99I	R0615L	CGWCG|	5	CpG	37	72.2
HpyCH4III	R0618L	ACN|GT	5	No	37	28.9
HpyCH4IV	R0619L	A|CGT	4	CpG	37	14.4
KpnI	R3142L	GGTAC|C	6	No	37	1.7
MluCI	R0538L	|AATT	4	No	37	6.9
MseI	R0525L	T|TAA	4	No	37	14.4
MspI	R0106L	C|CGG	4	No	37	1.4
NciI	R0196L	CC|SGG	5	CpG	37	3.4
NgoMIV	R0564L	G|CCGGC	6	CpG	37	6.9
NlaIII	R0125L	CATG|	4	No	37	14.4
NotI	R0189L	GC|GGCCGC	8	CpG	37	15.6
NsiI	R0127L	ATGCA|T	6	No	37	6.9
PsiI	R0657L	TTA|TAA	6	No	37	61.9
PstI	R3140L	CTGCA|G	6	No	37	0.7
RsaI	R0167L	GT|AC	4	CpG	37	6.3
SacI	R0156L	GAGCT|C	6	No	37	3.2
SacII	R0157L	CCGC|GG	6	CpG	37	3.3
SalI	R3138L	G|TCGAC	6	CpG	37	3.3
Sau96I	R0165L	G|GNCC	5	CpG,dcm	37	6.6
SbfI	R0642L	CCTGCA|GG	8	No	37	15.6
ScrFI	R0110L	CC|NGG	5	CpG,dcm	37	7.2
SgrAI	R0603L	CR|CCGGYG	8	CpG	37	7.2
SmaI	R0141L	CCC|GGG	6	CpG	25	3.3
SphI	R0182L	GCATG|C	6	No	37	14.4
SspI	R0132L	AAT|ATT	6	No	37	7.4
TaqI	R0149L	T|CGA	4	dam	65	1.6
TfiI	R0546L	G|AWTC	5	CpG	65	14.4
Tsp45I	R0583L	|GTSAC	5	No	65	37.2
XbaI	R0145L	T|CTAGA	6	dam	37	2.5
XhoI	R0146L	C|TCGAG	6	CpG	37	1.5

[a] The enzyme information, including methylation sensitive, active temperature and cost per unit (in Canadian dollars), was obtained from the catalogues of New England BioLabs (starting with R) and Clontech, or published literature (Jin *et al.*1994. Nucl. Acids Res. 22:3928–3929). Alternate name for the enzyme is given in parenthesis.
