# Supplementary material for: Increasing Genome Sampling and Improving SNP Genotyping for Genotyping-by-Sequencing with New Combinations of Restriction Enzymes
Source: G3 (Bethesda). 2016 Jan 27;6(4):845–56. doi: 10.1534/g3.115.025775 (PMC4825655; doi:10.1534/g3.115.025775)
Supplement: Supporting Information [file supp_g3.115.025775_TableS2.pdf]

Table S2 List of 22 species (8 plant, 13 animal and 1 fungus) and their genome sequence information obtained from NCBI database for *in silico* analysis of restriction enzyme digestions

Species	Scientific name	NCBI ID	SL[a]	NC [a]	Download link [a]	Reference [b]
Plant						
Arabidopsis	*Arabidopsis thaliana*	TAIR10	119,667,750	5	NCBI1/GCF_000001735.3	Meinke et al. 1998. Science 282:679-682
Black cottonwood	*Populus trichocarpa*	Genome ID 98	378,545,565	19	NCBI2/98	Tuskan et al. 2006. Science 313:1596-1604
Medicago	*Medicago truncatula*	MedtrA17_4.0	412,800,391	8	NCBI1/GCA_000219495.2	Tang et al. 2014. BMC Genomics 15:312
Wine grape	*Vitis vinifera*	Genome ID 401	426,176,009	19	NCBI2/401	Jaillon et al. 2007. Nature 449:463-467
Soybean	*Glycine max*	V1.1	973,779,290	20	NCBI1/GCF_000004515.3	Schmutz et al. 2010. Nature 463:178-183
Rice	*Oryza sativa japonica*	Build 4.0	382,778,125	12	NCBI1/GCF_000005425.2	Tanaka et al. 2008. Nucl Acids Res 36:D1028-33
Sorghum	*Sorghum bicolor*	Sorbi1	739,150,314	10	NCBI1/GCF_000003195.2	Paterson et al. 2009. Nature, 457:551-556
Maize	*Zea mays*	B73 RefGen_v3	2,067,622,303	10	NCBI1/GCF_000005005.1	Schnable et al. 2009. Science 326:1112-1115
Animal						
C. elegans	*Caenorhabditis elegans*	WBcel235	100,286,401	6	NCBI1/GCA_000002985.3	C. elegans Sequencing Consortium. 1998. Science. 282:2012-2018
Fruit fly	*Drosophila melanogaster*	Release 6 plus ISO1 MT	143,726,002	7	NCBI1/GCF_000001215.4/	Hoskins et al. 2007. Science 16:1625-1628
Honey bee	*Apis mellifera*	Amel_4.5	250,287,000	16	NCBI1/GCF_000002195.4	Elsik et al. 2014. BMC Genomics 15:86
Three-spined stickleback	*Gasterosteus aculeatus*	BROADS1	400,788,495	21	NCBI3/86302700	Jones et al. 2012. Nature 484:55-61
Northern pike	*Esox lucius*	EsoLuc1.0	877,830,608	25	NCBI1/GCF_000721915.1/	Rondeau et al. 2014. PLoS One 9: e102089
Zebra fish	*Danio rerio*	GRCz10	1,371,719,383	25	NCBI1/GCA_000002035.3	Howe et al. 2013. Nature. 496:498-503
Turkey	*Meleagris gallopavo*	Turkey_2.01	1,061,817,103	32	NCBI1/GCF_000146605.1	Dalloul et al. 2010. PLoS Biol 8: e1000475
Zebra finch	*Taeniopygia guttata*	Taeniopygia_guttata-3.2.4	1,232,135,591	35	NCBI1/GCA_000151805.2	Warren et al. 2010. Nature. 464:757-762
Dog	*Canus lupus familiaris*	CanFam3.1	2,410,976,875	39	NCBI1/GCA_000002285.2	Lindblad-Toh et al. 2005. Nature. 438:803-819
Housecat	*Felis sylvestris catus*	Felis_catus_8.0	2,641,342,258	19	NCBI1/GCA_000181335.3	Pontius et al. 2007. Genome Res. 17:1675-1689
House mouse	*Mus musculus*	GRCm38.p3	2,800,055,571	21	NCBI1/GCF_000001635.23/	Church et al. 2011. PLoS Biol 9:e1001091
Pigmy chimp	*Pan paniscus*	panpan1.1	3,286,643,896	24	NCBI1/GCA_000258655.2	Prufer et al. 2012. Nature. 486:527-531
Opossum	*Monodelphis domestica*	MonDom5	3,598,443,077	9	NCBI1/GCF_000002295.2	Mikkelsen et al. 2007. Nature 447:167-177
Fungi						
Baker's yeast	*Saccharomyces cerevisiae*	R64	12,157,105	16	NCBI1/GCA_000146045.2	Strope et al. 2015. Genome Res. 25:762-74

[a] SL=sequence length; NC=number of chromosomes; NCBI1=http://www.ncbi.nlm.nih.gov/assembly; NCBI2=http://www.ncbi.nlm.nih.gov/genome; NCBI3=http://www.ncbi.nlm.nih.gov/nuccore.

[b] The listed references here are independent from those used in the paper .
