# Supplementary material for: Increasing Genome Sampling and Improving SNP Genotyping for Genotyping-by-Sequencing with New Combinations of Restriction Enzymes
Source: G3 (Bethesda). 2016 Jan 27;6(4):845–56. doi: 10.1534/g3.115.025775 (PMC4825655; doi:10.1534/g3.115.025775)
Supplement: Supporting Information [file supp_g3.115.025775_TableS3.pdf]

Table S3 The *in silico* genome coverages (IgC; %) of 22 species with sequenced genomes (8 plant, 13 animals and 1 fungus) by DNA fragments of different ends and lengths (100-600bp) obtained from *in silico* digestions by 70 restriction enzyme pairs, including the GBS reference pair PstI+MspI

	Plant								Animal													Fungi			
Enzyme pair	Arabidopsis	Cottonwood	Medicago	Winegrape	Soybean	Rice	Sorghum	Maize	C. elegans	Fruit fly	Honey bee	Stickleback	Pike	Zebra fish	Turkey	Zebra finch	Dog	Housecat	House mouse	Pigmy chimp	Opossum	Baker's yeast	Mean_all	STD_all	Mean_plants
genome size (Mbp)	120	379	297	426	950	382	659	2060	83	158	220	401	377	1340	1040	1021	2328	2419	2726	3152	2998	12			
CviAII+HinfI	35.1	32.5	27.9	31.4	32.5	31.1	30.8	33.8	31.9	30.1	24.6	35.0	31.0	31.0	30.8	34.5	35.9	35.5	34.2	30.0	35.2	36.5	32.3	2.9	31.9
CviAII+DdeI	34.4	30.8	26.6	30.6	30.9	30.3	31.8	32.9	27.0	29.7	16.1	35.1	32.9	33.3	31.9	35.9	35.3	34.9	33.1	29.3	34.5	35.9	31.5	4.4	31.0
BfaI+HinfI	32.5	32.5	26.0	30.9	31.4	32.0	31.8	34.6	27.9	24.5	17.5	24.9	30.2	27.9	29.3	31.8	36.3	35.2	34.6	30.7	35.3	34.8	30.6	4.5	31.5
BfaI+DdeI	32.5	31.4	25.0	30.4	30.4	31.2	32.3	34.2	23.8	24.0	11.8	25.1	30.8	29.1	28.7	30.9	33.6	32.9	32.4	29.0	33.6	34.4	29.4	5.1	30.9
CviAII+TfiI	33.4	27.9	24.3	27.2	27.7	25.8	26.2	27.6	29.3	25.8	24.5	26.6	23.2	23.7	24.7	26.6	28.4	29.7	25.9	24.0	30.8	33.9	27.2	2.9	27.5
BfaI+TfiI	31.8	29.1	23.6	27.9	28.2	27.0	27.3	29.1	25.9	21.4	17.8	20.5	24.0	22.4	24.4	25.8	30.0	31.8	28.0	25.6	30.9	32.7	26.6	3.9	28.0
MluCI+HinfI	25.3	18.0	14.1	19.8	19.2	24.9	28.1	29.1	13.5	20.7	14.1	33.0	28.4	24.2	25.1	26.6	29.6	29.4	30.1	23.5	24.5	24.2	23.9	5.5	22.3
MluCI+DdeI	24.4	17.3	13.6	19.4	18.6	24.0	27.5	29.0	10.2	19.6	7.4	33.3	30.1	26.0	26.5	28.4	28.6	29.4	29.2	23.5	24.1	22.3	23.3	6.7	21.7
CviAII+ApeKI	19.3	17.2	12.9	13.4	14.2	25.3	22.6	23.8	18.5	27.7	11.0	33.7	25.6	26.6	28.1	31.8	24.9	23.9	25.9	21.0	20.9	25.4	22.4	6.0	18.6
HinfI+MseI	28.1	22.0	18.7	24.7	24.3	28.0	28.8	30.6	27.0	7.9	4.3	10.4	9.8	21.6	8.4	28.3	28.8	28.7	11.2	26.1	7.0	31.6	20.7	9.2	25.6
HinfI+HpyCH4IV	29.4	22.7	21.6	18.0	23.1	27.9	27.2	27.9	30.5	14.3	11.6	16.1	14.8	24.8	11.7	16.0	19.4	22.7	9.3	15.8	5.6	35.4	20.3	7.7	24.7
DdeI+MseI	27.1	20.8	17.9	24.1	23.2	26.9	29.3	30.3	21.5	8.3	3.7	10.2	9.3	22.6	6.0	29.5	29.0	29.1	7.5	25.1	5.5	30.0	19.9	9.5	25.0
MluCI+TfiI	23.7	15.0	11.8	16.6	15.9	19.9	23.3	24.6	11.8	16.9	13.1	24.8	21.0	18.4	19.6	19.8	22.7	25.3	22.6	18.5	20.4	20.6	19.4	4.1	18.9
DdeI+HpyCH4IV	29.2	22.2	20.8	17.5	22.6	27.2	27.2	27.5	26.3	14.9	9.3	15.9	13.8	26.0	8.6	14.6	18.0	20.7	6.5	14.2	4.7	35.2	19.2	8.1	24.3
ApeKI+BfaI	19.5	18.3	13.1	14.9	15.1	26.3	23.3	24.3	16.8	12.6	5.9	13.8	14.7	25.2	13.5	29.8	26.6	23.9	14.1	22.8	10.5	24.3	18.6	6.3	19.4
TfiI+HpyCH4IV	29.0	20.7	19.7	16.8	21.5	24.2	24.2	23.7	28.3	13.9	12.3	15.2	13.2	20.2	10.9	13.3	16.8	20.7	9.1	13.8	6.1	33.3	18.5	6.9	22.5
NlaIII+MluCI	25.7	19.4	16.0	20.2	19.6	25.4	27.0	29.0	13.4	5.2	2.4	10.8	7.7	27.1	6.3	28.7	29.3	30.2	8.0	24.3	5.4	24.3	18.4	9.4	22.8
TfiI+MseI	26.5	19.0	16.3	21.5	20.9	23.6	24.3	26.3	24.5	7.8	5.0	9.7	8.5	16.0	7.7	21.4	22.2	23.9	10.1	21.0	7.1	28.3	17.8	7.5	22.3
CviAII+AvaII	15.6	14.0	12.3	14.5	14.6	18.7	20.8	24.7	12.4	15.9	7.0	22.9	19.6	13.4	14.5	17.7	20.4	20.9	22.3	14.9	20.0	18.1	17.1	4.2	16.9
ApeKI+MseI	17.2	14.1	9.9	13.2	12.8	25.1	22.9	23.2	15.8	7.2	3.8	10.0	9.5	19.5	7.0	26.7	21.8	20.4	10.8	19.2	6.2	21.3	15.3	6.8	17.3
AvaII+BfaI	16.1	15.3	12.3	15.8	15.5	20.6	21.8	26.2	11.3	9.0	3.9	11.6	12.3	13.0	9.0	17.3	22.5	21.4	13.4	16.2	10.0	18.1	15.1	5.3	17.9
HinP1I+HinfI	11.2	10.0	7.7	8.0	10.6	22.3	22.3	25.6	19.2	26.6	20.0	26.9	16.5	17.8	7.8	7.2	10.4	12.8	8.3	8.6	4.1	23.8	14.9	7.3	14.7
HhaI+HinfI	11.2	10.0	7.7	8.0	10.6	22.3	22.3	25.6	19.2	26.6	20.0	26.9	16.5	17.8	7.8	7.2	10.4	12.8	8.3	8.6	4.1	23.8	14.9	7.3	14.7
ApeKI+HpyCH4IV	17.4	13.7	11.0	9.2	11.4	23.0	20.5	21.6	18.0	13.4	6.5	15.9	13.6	22.3	10.3	14.4	15.5	17.5	8.9	12.3	4.6	25.7	14.8	5.5	16.0
HinfI+MspI	19.0	13.3	11.3	11.2	11.8	24.0	23.3	26.6	22.7	12.5	8.3	14.6	11.3	15.8	4.6	8.5	14.6	14.0	6.8	11.4	3.0	23.8	14.2	6.6	17.6
HhaI+DdeI	11.4	9.7	7.6	7.7	10.6	21.7	22.1	23.9	16.4	26.2	12.6	26.6	16.5	18.2	6.8	6.4	10.2	10.5	6.3	6.9	3.3	22.8	13.8	7.3	14.3
HinP1I+DdeI	11.4	9.7	7.6	7.7	10.6	21.7	22.1	23.9	16.4	26.2	12.6	26.6	16.5	18.2	6.8	6.4	10.2	10.5	6.3	6.9	3.3	22.8	13.8	7.3	14.3
DdeI+MspI	19.7	13.3	11.3	11.0	12.0	23.7	23.6	27.5	20.5	13.2	7.4	14.6	10.6	15.9	3.2	7.8	13.9	12.5	4.4	9.5	2.1	24.2	13.7	7.0	17.8
ApeKI+MluCI	15.5	10.9	7.1	9.9	10.1	22.0	20.8	21.8	7.7	4.8	2.1	12.4	10.0	21.6	6.6	25.2	21.9	20.2	10.3	17.8	4.8	15.5	13.6	6.9	14.8
HinP1I+TfiI	11.6	9.3	7.4	7.7	10.3	19.8	20.1	22.1	18.0	23.3	20.4	22.3	13.6	14.7	6.5	5.9	9.4	11.9	7.0	7.6	3.8	22.3	13.4	6.5	13.5
HhaI+TfiI	11.6	9.3	7.4	7.7	10.3	19.7	20.1	22.1	18.0	23.3	20.4	22.3	13.6	14.7	6.5	5.9	9.4	11.9	7.0	7.6	3.8	22.3	13.4	6.5	13.5
TfiI+MspI	19.5	12.4	10.4	10.8	10.9	21.0	20.8	22.9	21.4	12.3	9.6	14.2	10.3	13.2	4.3	7.0	13.1	12.9	6.8	10.2	3.4	22.9	13.2	5.9	16.1
AvaII+MseI	12.9	9.8	7.9	12.6	12.0	18.1	20.4	23.6	10.5	6.1	2.9	9.3	8.4	9.8	5.9	16.2	18.2	18.1	10.1	13.8	5.6	15.5	12.2	5.3	14.6
AvaII+HpyCH4IV	14.4	11.3	10.4	10.2	12.0	17.7	18.7	21.9	12.1	9.4	4.3	13.2	11.5	12.0	7.3	9.6	13.9	15.8	8.3	9.3	4.5	18.3	12.1	4.5	14.6
AvaII+ApeKI	10.4	9.7	7.0	9.1	8.9	17.7	17.4	20.2	7.8	8.2	2.9	12.8	11.4	12.8	7.8	17.1	18.6	17.3	12.8	13.3	8.5	14.4	12.1	4.5	12.5
MspI+MseI	16.6	8.8	7.9	9.5	9.5	21.6	22.0	24.3	19.5	7.6	4.7	9.8	7.8	12.5	3.3	7.8	12.3	12.2	6.1	11.1	2.3	20.0	11.7	6.3	15.0
HinP1I+ApeKI	7.6	6.3	4.3	4.7	5.9	17.1	16.3	17.7	11.5	23.6	8.8	24.8	13.8	15.4	6.1	5.5	8.8	10.5	7.0	7.1	2.6	17.3	11.0	6.3	10.0
AvaII+MluCI	11.5	7.8	5.9	9.9	9.7	16.4	19.0	22.4	5.0	4.4	1.7	11.3	8.8	11.2	5.7	15.1	18.8	18.5	9.7	12.8	5.0	10.8	11.0	5.5	12.8
ApeKI+MspI	12.4	8.6	6.3	6.8	6.5	18.8	17.0	19.5	14.1	11.2	5.2	14.1	10.2	14.1	3.4	7.1	12.0	11.6	6.6	9.7	2.5	18.0	10.7	4.9	12.0
ApeKI+HhaI	7.7	6.4	4.4	4.7	5.9	17.1	16.3	17.5	11.5	12.1	5.3	13.5	8.7	15.6	3.5	5.7	9.0	10.6	4.5	7.2	1.7	17.3	9.4	5.0	10.0
HinP1I+AvaII	6.4	5.3	4.2	5.1	6.8	14.3	16.2	19.9	8.0	14.2	6.1	18.8	11.7	8.7	4.7	4.7	8.7	9.4	6.5	5.8	2.8	13.1	9.2	4.9	9.8
AvaII+MspI	9.9	6.7	5.7	6.7	6.9	14.7	16.6	19.1	9.3	7.7	3.3	11.5	8.4	7.6	3.1	5.5	11.1	10.6	6.3	7.6	2.6	13.1	8.8	4.3	10.8
NsiI+BfaI	10.3	12.7	10.9	12.3	13.0	12.0	12.1	9.7	6.1	5.0	3.4	5.3	6.4	11.6	6.4	9.7	8.9	8.3	5.7	7.8	5.1	8.4	8.7	2.9	11.6
AvaII+HhaI	6.4	5.3	4.2	5.1	6.8	14.3	16.2	19.9	8.0	8.4	3.7	11.5	7.6	8.7	3.2	4.7	8.7	9.4	4.3	5.8	1.8	13.1	8.1	4.5	9.8
HindIII+BfaI	13.6	10.9	8.3	11.6	10.6	8.0	10.1	9.5	9.1	4.1	2.7	4.1	3.9	7.8	6.3	10.2	8.4	8.4	5.4	7.5	4.9	10.3	8.0	2.9	10.3
NsiI+HpyCH4IV	9.5	9.2	9.2	7.3	9.9	10.8	10.7	8.5	6.6	5.3	3.8	6.1	6.0	10.1	4.9	4.8	5.1	5.6	3.4	4.3	2.0	8.8	6.9	2.6	9.4
EcoRI+BfaI	8.5	8.2	6.5	7.9	7.7	6.3	6.8	7.0	10.6	4.2	4.6	2.9	3.5	4.7	4.6	7.9	7.8	8.2	5.3	6.8	6.3	9.9	6.6	2.0	7.4
HindIII+HpyCH4IV	12.3	8.0	6.8	6.9	8.0	7.1	9.4	7.4	10.2	4.8	3.2	4.8	3.7	7.1	4.9	5.2	5.1	5.9	3.3	4.1	1.9	10.7	6.4	2.7	8.2
NsiI+MseI	7.0	7.1	6.1	8.2	8.3	9.0	9.9	8.4	4.9	2.6	1.1	3.3	3.3	6.4	3.1	6.4	5.5	5.3	3.4	5.0	2.0	6.3	5.6	2.4	8.0
NsiI+MspI	6.5	5.5	5.2	4.7	5.6	9.7	10.1	8.5	5.1	5.1	2.8	6.0	4.7	6.8	1.8	2.3	3.9	3.3	2.5	3.0	1.0	6.3	5.0	2.4	7.0
AflII+BfaI	7.1	6.1	5.5	7.6	7.2	4.5	5.8	6.4	2.3	3.3	1.3	2.5	2.8	4.6	4.0	6.2	6.9	6.7	4.0	5.7	4.0	4.9	5.0	1.8	6.3
NsiI+MluCI	6.0	5.7	4.5	6.2	6.3	7.4	8.7	7.5	2.3	1.6	0.6	4.2	3.6	8.0	2.9	6.0	5.7	5.4	3.4	4.8	1.5	4.5	4.8	2.2	6.5
HindIII+MspI	8.9	4.8	3.9	4.9	4.4	6.1	8.4	7.9	8.0	4.4	2.6	4.6	2.9	4.7	1.8	2.7	4.0	3.5	2.4	3.0	1.1	7.6	4.7	2.3	6.2
EcoRI+MseI	6.4	5.0	4.2	5.6	5.3	5.3	5.7	5.6	9.0	2.2	2.2	2.0	1.8	2.9	2.2	6.1	5.3	5.8	3.2	4.8	3.3	7.9	4.6	2.0	5.4
SphI+BfaI	3.3	5.6	3.3	4.6	5.5	7.8	7.0	5.2	2.5	3.2	1.2	3.3	3.6	5.6	3.9	5.1	4.4	4.2	3.8	4.9	1.9	3.4	4.2	1.6	5.3
SacI+MseI	4.5	2.6	1.5	3.5	2.5	7.1	7.5	9.9	5.3	2.1	0.8	3.5	2.3	3.7	2.8	7.3	5.2	5.2	4.4	4.4	1.9	2.9	4.1	2.3	4.9
PstI+MspI	3.5	2.3	1.6	1.8	1.5	5.6	6.0	5.5	3.1	4.5	0.6	8.9	4.8	6.2	3.3	5.3	5.4	4.6	3.0	5.0	0.8	4.4	4.0	2.0	3.5
EcoRI+MspI	5.7	3.8	3.2	3.2	3.0	5.1	6.2	5.2	9.1	4.3	4.7	3.3	2.6	2.8	1.3	2.2	3.6	3.4	2.1	2.8	1.3	7.3	3.9	1.9	4.4
AflII+HpyCH4IV	6.6	4.4	4.5	4.6	5.2	3.9	5.3	5.5	2.7	3.8	1.6	2.9	2.7	4.3	3.2	3.1	4.0	4.6	2.6	3.2	1.8	5.1	3.9	1.3	5.0
EcoRI+MluCI	5.6	3.7	2.9	4.1	4.1	4.1	5.2	5.1	3.7	1.6	1.2	2.5	2.0	3.2	2.2	5.7	5.3	6.0	2.9	4.5	2.6	5.7	3.8	1.5	4.3
SphI+HpyCH4IV	3.0	4.2	2.7	3.0	4.2	7.1	6.3	5.0	2.7	3.4	1.2	3.7	3.3	5.1	3.2	2.7	2.7	3.0	2.5	2.8	0.8	4.1	3.5	1.5	4.4
SphI+MseI	2.4	3.7	2.1	3.7	3.9	6.6	6.6	5.1	2.2	2.2	0.7	2.6	2.2	4.0	2.4	4.0	3.3	3.2	2.6	4.2	1.1	3.1	3.3	1.5	4.2
SphI+MluCI	2.1	2.8	1.5	2.8	3.1	5.7	5.9	4.8	1.1	1.5	0.4	3.1	2.4	4.5	2.3	3.7	3.4	3.4	2.8	3.6	0.9	2.2	2.9	1.4	3.6
AflII+MluCI	4.4	2.7	2.3	3.9	3.3	2.7	4.3	4.9	0.9	1.2	0.3	2.1	1.6	3.2	1.8	3.8	4.5	4.6	2.4	3.5	1.4	2.5	2.8	1.3	3.6
AflII+MspI	4.7	2.6	2.6	2.7	2.5	3.4	5.3	6.3	2.1	3.6	1.3	2.9	2.1	2.8	1.1	1.5	3.0	2.7	1.8	2.3	0.9	3.6	2.8	1.3	3.8
SphI+MspI	2.1	2.6	1.6	2.0	2.6	5.9	5.4	4.9	2.2	3.0	1.0	3.5	2.6	3.4	1.3	1.4	2.2	2.0	1.9	2.2	0.5	2.9	2.6	1.4	3.4
KpnI+MseI	1.8	1.2	1.4	1.9	2.4	2.9	3.5	5.9	1.7	0.7	0.3	1.1	1.3	0.9	0.9	2.4	2.2	2.3	1.6	2.2	0.8	4.1	2.0	1.3	2.6
AflII+MseI	2.7	1.7	1.6	2.7	2.3	1.7	2.4	2.5	0.9	0.9	0.2	0.9	0.8	1.4	1.0	2.2	2.3	2.4	1.3	2.1	0.9	1.9	1.7	0.7	2.2
SphI+EcoRI	1.0	1.7	1.0	1.4	1.5	1.7	1.5	1.5	1.1	1.1	0.7	0.8	0.8	1.0	1.2	1.4	1.1	1.1	1.2	1.2	0.9	1.2	1.2	0.3	1.4
SbfI+EcoRI	0.1	0.1	0.0	0.0	0.0	0.1	0.1	0.1	0.0	0.1	0.0	0.2	0.1	0.1	0.2	0.4	0.3	0.2	0.1	0.2	0.1	0.1	0.1	0.1	0.1
